# Functional and cognitive capacity differ in dystonic motor subtypes when compared to choreatic and hypokinetic‐rigid motor subtypes in Huntington's disease

**DOI:** 10.1002/brb3.1704

**Published:** 2020-06-12

**Authors:** Jannis Achenbach, Sarah Maria von Hein, Carsten Saft

**Affiliations:** ^1^ Department of Neurology St. Josef‐Hospital Bochum Germany

**Keywords:** dystonia, Huntington's disease, movement disorders

## Abstract

**Background:**

Motor phenotypes in Huntington's disease vary manifold. Phenotype classification is essential to adapt treatment. The aim of this study was to classify a dystonic subtype closer.

**Methods:**

A total of 7,512 manifest ENROLL‐HD participants were subdivided into mainly choreatic (*N* = 606), dystonic (*N* = 402), and hypokinetic‐rigid (*N* = 369) subjects. Cognitive (verbal fluency, symbol digit, stroop color, trail making, Mini‐Mental State Examination), functional (total functional capacity, Independence Scale), and psychiatric (problem behaviors assessment, Hospital Anxiety and Depression Scale) performance was evaluated at baseline visit.

**Results:**

Symptoms onset for dystonic were similar to hypokinetic‐rigid, but earlier compared to choreatic subjects (*p* < .001). Cognition was better in both groups compared to hypokinetic rigid (all *p* < .001). Functionality differed between all groups (all *p* < .001). Differences remained (all *p* < .001) after controlling for CAP score, CAG, age, disease duration, and education.

**Conclusions:**

Motor subtypes differ in functional and cognitive capacities but less in psychiatric. We identified better cognitive and functional capacities and similar onsets in predominant dystonic compared to hypokinetic‐rigid patients.

## Graphical abstract

The research confirmed that motoric phenotypes are relevant for functional and cognitive capacities in HD. Onsets in dystonic patients are similar to hypokinetic‐rigids. Cognitive capacity is better in dystonic and choreatic than hypokinetic‐rigids patients.

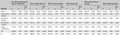

## 1 INTRODUCTION

Neurodegenerative Huntington's disease (HD) is caused by CAG trinucleotide expansion in the huntingtin gene (HTT; Walker, 2007). Symptomatic manifestation accompanies with heterogeneous motoric, cognitive, and psychiatric symptoms. Although dominant motor symptoms are hyperkinetic, they can vary manifold with bradykinesia, dystonia, myoclonus, or as hypokinetic‐rigid (Roos, 2010; Saft, Lauter, Kraus, Przuntek, & Andrich, 2006).

Several studies investigated different aspects of bradykinesia, respectively, hypokinetic‐rigidity or chorea in HD (Girotti, Marano, Soliveri, Geminiani, & Scigliano, 1988; Mahant, McCusker, Byth, & Graham, 2003; Reedeker, Van Der Mast, Giltay, Van Duijn, & Roos, 2010; Sanchez‐Pernaute et al., 2000; Thompson et al., 1988; Young et al., 1986). More recently, Hart et al. (2013) focused on aspects of predominant choreatic versus hypokinetic‐rigid motor phenotypes and described a better capacity for global and cognitive functioning of their choreatic group.

So far only a few smaller studies investigated the role of dystonia in HD (Mahant et al., 2003; Zande et al., 2017). Zande et al. (2017) described a high prevalence of dystonia as being a late symptom correlating with increasing HD stage and disease duration. In addition, a strong relationship with functional status of dystonia and rigidity was described (Carlozzi et al., 2019). To our knowledge, no study compared three different phenotypes with predominant dystonia, chorea, and hypokinetic‐rigidity which are essential for symptomatic treatments (Saft, von Hein, & Lucke, 2018; Wojtecki, Groiss, & Ferrea, 2015; Zittel et al., 2018). For a better understanding, we investigated whether there are collectives in the European Huntington's Disease Network ENROLL‐HD study classified as predominant choreatic, dystonic, and hypokinetic‐rigid in order to compare cognitive, functional, and psychiatric performance data (Landwehrmeyer, Fitzer‐Attas, & Giuliano, 2017). We hypothesize a better cognitive functioning of a choreatic and dystonic group when compared to a hypokinetic‐rigid group and possibly more depression due to pain in our dystonic subgroup.

## 2 PATIENTS AND METHODS

A total of 7,512 subjects (>18 years) with manifest HD (diagnostic confidence level: 4; >36 CAG; total motor score (TMS): >5) participating in the ENROLL‐HD study were analyzed (Kieburtz et al., 1996).

Enroll‐HD is a global clinical research platform designed to facilitate clinical research in Huntington's disease. Core datasets are collected annually from all research participants as part of this multicentre longitudinal observational study. Data are monitored for quality and accuracy using a risk‐based monitoring approach. All sites are required to obtain and maintain local ethical approval.

For subdividing predominant motor phenotypes, sum scores were calculated according to Hart et al. (2013) Scores had to differ by at least one standard deviation compared to the entire cohort. Classification of predominant choreatic (*N* = 606) due to chorea scale and hypokinetic‐rigid (*N* = 369) due to hypokinetic‐rigidity with finger taps, pronate–supinate hands, bradykinesia, and rigidity, phenotypes were identified in UHDRS motor assessment (Hart et al., 2013). Equally onset and classification of dystonia were evaluated due to items for dystonia in trunk, right, and left upper and lower extremities as part of the UHDRS motor score, and (*N* = 402) subjects were classified as predominant dystonic. Interference with predominant hypokinetic‐rigidity and dystonia (*n* = 661) or smaller deviations were regarded as mixed phenotypes (*n* = 6,135) and excluded.

Differences among group means were assessed using ANOVA with post hoc Tukey's HSD tests in IBM SPSS Statistics V.25. Afterward, one‐way ANCOVAs were conducted determining differences between subtypes on functional and cognitive assessments controlling for CAP score (Zhang et al., 2011), CAG, age, disease duration, and education.

## 3 RESULTS

Significant mean differences were detected between choreatic, dystonic, and hypokinetic‐rigid groups regarding all demographic parameters (all *p* < .001) (Table 1). Post hoc comparisons indicated CAP score was higher, education level lower, and disease duration longer in hypokinetic compared to dystonic and choreatic group (all *p* < .001). The CAG mean was lower in the choreatic versus dyston and hypokinetic group (all *p* < .001). Comparison of age indicated that the dystonic group was younger than choreatic and hypokinetic‐rigid (all *p* < .001).

**TABLE 1:** Analysis of demographics, motoric, cognitive, functional, and psychiatric capacities in different motor subtypes

	Choreatic (1) *N* = 606	Dyston (2) *N* = 402	Hypokinetic‐rigid (3) *N* = 369	*F*	*p*	Part. *η* ^2^	*p* (1) versus (2)	*p* (1) versus (3)	*p* (2) versus (3)
Demographic parameters									
Age (years); *M* (*SD*)	56.1 (12.0)	51.2 (13.5)	55.8 (14.2)	14.10	<.001	0.020	<.001	.929	<.001
CAG high	43.6 (3.4)	45.3 (5.3)	45.4 (5.8)	23.87	<.001	0.034	<.001	<.001	.974
Disease duration (years)	8.6 (5.3)	7.9 (5.5) *n* = 400	10.3 (5.0) *n* = 361	20.96	<.001	0.030	.054	<.001	<.001
Education level (ISCED 0–6)	3.4 (1.2)	3.4 (1.2) *n* = 399	3.0 (1.3) *n* = 366	13.92	<.001	0.020	.874	<.001	<.001
CAP score	526.2 (83.2)	548.1 (92.1)	589.4 (110.4)	52.15	<.001	0.071	<.005	<.001	<.001
Timed onsets									
Age of onset motor symptoms (years)	47.5 (11.6)	44.0 (13.8) *n* = 400	45.6 (14.1) *n* = 361	8.84	<.001	0.013	<.001	.072	.216
Rater's estimate of symptom onset (years)	47.25 (11.9) *n* = 572	43.2 (13.2) *n* = 378	44.8 (13.5) *n* = 340	12.18	<.001	0.019	<.001	.013	.215
Date of clinical HD diagnosis (years)	51.2 (12.0) *n* = 601	46.7 (13.6) *n* = 396	48.6 (13.9) *n* = 362	14.71	<.001	0.021	<.001	.009	.102
Symptoms first noticed by participant (years)	48.3 (12.1) *n* = 577	44.8 (13.4) *n* = 374	45.8 (13.8) *n* = 338	9.09	<.001	0.014	<.001	.014	.566
Symptoms first noticed by family (years)	47.2 (11.9) *n* = 564	43.5 (13.5) *n* = 375	45.2 (13.7) *n* = 343	9.41	<.001	0.014	<.001	.072	.163
At what age did cognitive impairment first start to have an impact on daily life? (years)	50.1 (12.4) *n* = 381	46.2 (13.9) *n* = 274	48.8 (14.5) *n* = 302	6.76	<.005	0.014	<.005	.442	.050
At what age did perseverative obsessive behavior begin? (years)	52.1 (13.5) *n* = 333	48.1 (14.4) *n* = 221	49.9 (14.3) *n* = 219	5.76	<.005	0.015	<.005	.167	.340
At what age did irritability begin? (years)	47.9 (13.3) *n* = 413	44.2 (14.6) *n* = 255	47.6 (15.5) *n* = 262	5.86	<.005	0.012	<.005	.951	.020
At what age did depression begin? (years)	47.1 (13.5) *n* = 427	42.1 (14.4) *n* = 298	45.5 (14.8) *n* = 264	10.95	<.001	0.022	<.001	.337	.012
At what age did apathy begin? (years)	51.8 (13.1) *n* = 386	46.8 (14.2) *n* = 251	51.1 (14.7) *n* = 268	10.92	<.001	0.024	<.001	.780	<.005
At what age did violent or aggressive behavior begin? (years)	47.7 (14.4) *n* = 223	44.3 (14.8) *n* = 134	47.2 (16.1) *n* = 189	2.26	.105	0.008	.101	.939	.210
At what age did psychosis (hallucinations or delusions) begin? (years)	49.6 (12.7) *n* = 61	45.9 (15.6) *n* = 60	49.9 (15.8) *n* = 93	1.47	.231	0.014	.359	.992	.239
Motoric parameter									
TMS (UHDRS)	50.4 (11.4)	52.2 (10.3)	63.5 (10.7)	180.33	<.001	0.208	.028	<.001	<.001
Cognitive parameters									
Verbal fluency test (letters): Total correct (in 60 s)	10.7 (4.7) *n* = 595	9.9 (4.6) *n* = 394	5.7 (3.8) *n* = 319	136.85	<.001	0.173	.021	<.001	<.001
Verbal fluency test (letters): Total correct (in 180 s)	18.1 (10.5) *n* = 472	16.1 (9.4) *n* = 295	8.0 (6.4) *n* = 183	75.66	<.001	0.138	<.005	<.001	<.001
SDMT: total correct (in 90 s)	19.3 (10.3) *n* = 555	16.7 (9.0) *n* = 377	7.0 (7.4) *n* = 257	154.71	<.001	0.207	<.001	<.001	<.001
Stroop color naming test: total correct (in 45 s)	37.3 (14.1) *n* = 592	35.5 (14.1) *n* = 387	20.2 (13.7) *n* = 311	164.78	<.001	0.203	.113	<.001	<.001
Stroop word reading test: total correct (in 45 s)	49.6 (18.6) *n* = 581	46.3 (18.2) *n* = 389	25.8 (18.2) *n* = 297	173.35	<.001	0.215	.016	<.001	<.001
Stroop interference test: total correct (in 45 s)	20.3 (9.8) *n* = 512	18.9 (8.6) *n* = 330	10.1 (8.2) *n* = 198	91.84	<.001	0.150	.076	<.001	<.001
Trail making test: Part A time to complete (in seconds)	82.7 (51.0) *n* = 437	91.9 (54.1) *n* = 271	151.4 (73.4) *n* = 126	75.08	<.001	0.153	.084	<.001	<.001
Trail making test: Part B time to complete (in seconds)	173.0 (68.1) *n* = 415	188.9 (63.7) *n* = 253	223.0 (45.7) *n* = 116	28.27	<.001	0.068	.005	<.001	<.001
MMSE: total correct	24.5 (4.0) *n* = 409	23.3 (4.0) *N* = 253	17.8 (6.1) *n* = 156	128.42	<.001	0.240	.005	<.001	<.001
Functional parameters									
TFC (UHDRS)	7.3 (2.9)	6.6 (3.1) *n* = 401	3.2 (2.6) *n* = 368	242.07	<.001	0.261	<.005	<.001	<.001
IS (%)	73.7 (12.8)	70.2 (14.2) *n* = 400	51.0 (17.5)	295.26	<.001	0.301	<.005	<.001	<.001
Psychiatric parameters									
PBA‐s: sum score apathy	3.6 (4.3) *n* = 604	3.9 (4.5) *n* = 400	6.6 (5.8) *n* = 358	49.71	<.001	0.068	.604	<.001	<.001
PBA‐s: sum score executive function	3.8 (5.8) *n* = 599	4.3 (6.1) *n* = 399	5.7 (7.1) *n* = 348	9.93	<.001	0.015	.463	<.001	.007
PBA‐s: sum score depression	4.9 (6.5) *n* = 603	5.0 (6.0) *n* = 401	4.8 (6.2) *n* = 356	0.093	.911	0.000	.981	.956	.903
PBA‐s: sum score irritability, aggression	3.5 (5.1) *n* = 604	2.9 (4.7) *n* = 401	4.1 (6.2) *n* = 368	4.90	.008	0.007	.199	.190	.05
PBA‐s: sum score psychosis	0.4 (2.2) *n* = 603	0.4 (1.9) *n* = 398	0.5 (2.4) *n* = 355	0.516	.597	0.001	.984	.665	.617
HADS‐SIS: depression subscore	6.2 (3.9) *n* = 386	6.6 (4.4) *n* = 200	7.4 (4.6) *n* = 135	3.96	.019	0.011	.511	.014	.226
HADS‐SIS: irritability subscore	6.0 (4.5) *n* = 385	5.7 (5.0) *n* = 200	5.0 (4.1) *n* = 134	2.42	.090	0.007	.716	.073	.360
HADS‐SIS: outward irritability subscore	3.6 (2.7) *n* = 387	3.3 (2.8) *n* = 201	3.3 (2.8) *n* = 135	0.776	.461	0.002	.553	.599	.998
HADS‐SIS: inward irritability subscore	2.4 (2.4) *n* = 387	2.4 (2.9) *n* = 201	1.7 (2.1) *n* = 135	4.36	.013	0.012	.974	.011	.042
HADS‐SIS: anxiety subscore	6.0 (4.2) *n* = 387	6.0 (4.7) *n* = 200	5.2 (3.8) *n* = 132	1.94	.144	0.005	.989	.133	.239

Note

Statistical data given as a comparison of mean values (standard deviation) between choreatic, dystonic, and hypokinetic‐rigid groups using ANOVA and post hoc tests (Tukey's HSD).

Abbreviations: *F*, *F* value; HADS‐SIS, Hospital Anxiety and Depression Scale; IS, Independence Scale; M, mean; MMSE, Mini‐Mental State Examination; *p*, *p* value; Part *η* ^2^, effect size; PBA‐s, problem behaviors assessment; *SD*, standard deviation; SDMT, symbol digit modality test; TFC, total functional score; TMS, total motor score.

Analysis of motoric manifestation verified a higher UHDRS‐TMS for hypokinetic subjects when compared to other groups (all *p* < .001). The dystonic group had earlier motoric onset when compared to choreatic (*p* < .001) but not to hypokinetic‐rigid subjects (*p* = .216). Comparisons of choreatic versus hypokinetic did not differ in means (*p* = .072). Timed onsets for the rater's estimation date of HD diagnosis and symptoms noticed by participant and family differed in same manner as described for motoric manifestation.

Differences regarding cognitive capacity revealed lower performance of hypokinetic if compared to choreatic or dystonic participants (all *p* < .001). Differences of the choreatic versus dystonic group were not significant except for better performance in SDMT (*p* < .001) and verbal fluency (180 s; *p* < .005).

Functional capacities differed for the UHDRS‐total functional capacity (TFC) score and Independence Scale (IS) (all *p* < .001) detecting choreatic with better capacities compared to dystonic (all *p* < .005) and hypokinetic (all *p* < .001). Post hoc tests indicated better functionality for the dystonic versus the hypokinetic group (all *p* < .001).

Beside higher apathy scores for hypokinetic versus. dystonic and choreatic (all *p* < .001) and executive function (choreatic vs. hypokinetic; *p* < .001), the psychiatric scores revealed no group differences.

One‐way ANCOVAs determined effects in cognitive and functional scores remained significant (all *p* < .001) after controlling for covariates (Table 2). Controlling for the covariate CAP score indicated highest effect‐ changes (in partial *η* ^2^) compared to other covariates.

**TABLE 2:** Analysis of cognitive and functional capacities in choreatic, dystonic, and hypokinetic‐rigid motor subtypes controlling for covariates

ANCOVA	Mot. Phenotype (choreatic, dystonic, hypokinetic)			With covariate CAP score			With covariate CAG high			With covariate age			With covariate disease duration			With covariate education		
	*F*	*p*	Part. *η* ^2^	*F*	*p*	Part. *η* ^2^	*F*	*p*	Part. *η* ^2^	*F*	*p*	Part. *η* ^2^	*F*	*p*	Part. *η* ^2^	*F*	*p*	Part. *η* ^2^
SDMT	154.71	<.001	0.207	128.26	<.001	0.178	156.44	<.001	0.209	156.73	<.001	0.209	142.24	<.001	0.195	141.51	<.001	0.193
Verbal fluency (letters)	136.85	<.001	0.173	111.95	<.001	0.147	136.60	<.001	0.173	137.38	<.001	0.174	124.81	<.001	0.162	124.64	<.001	0.161
Stroop color naming	164.28	<.001	0.203	141.91	<.001	0.181	172.56	<.001	0.212	166.38	<.001	0.206	150.18	<.001	0.190	150.43	<.001	0.190
Stroop word reading	173.35	<.001	0.215	145.63	<.001	0.187	176.54	<.001	0.218	175.23	<.001	0.217	157.69	<.001	0.201	158.29	<.001	0.201
Stroop interference	91.84	<.001	0.150	82.05	<.001	0.137	104.12	<.001	0.167	98.47	<.001	0.160	83.24	<.001	0.139	82.04	<.001	0.137
TFC	242.08	<.001	0.261	202.88	<.001	0.228	240.65	<.001	0.260	242.69	<.001	0.261	215.31	<.001	0.240	225.16	<.001	0.249
IS	295.27	<.001	0.301	248.82	<.001	0.266	294.40	<.001	0.300	296.50	<.001	0.302	266.36	<.001	0.281	278.75	<.001	0.290

Note

Comparison of mean values.

Abbreviations: *F*, *F* value; IS, Independence Scale; *p*, *p* value; Part *η* ^2^, effect size; SDMT, symbol digit modality test; TFC, total functional score.

## 4 DISCUSSION

To our knowledge, this is the first study comprising data coming from a large cohort of manifest HD patients analyzing subjects with motor symptoms in terms of predominantly dystonia compared to choreatic and hypokinetic‐rigid regarding their cognitive, functional, and psychiatric performance.

If compared to hypokinetic‐rigids, no differences regarding onsets were observed in the dystonic group except for earlier apathy. Contrary dystonic participants showed earlier onsets compared to choreatic. In contrast to earlier publication, no difference was observed for the choreatic compared to the hypokinetic‐rigid group even if CAG repeats and the CAP score were significantly lower in the choreatic group (Hart et al., 2013). It is reasonably possible that in previous research, dystonic patients were implemented partly within a hypokinetic‐rigid cohort. As mentioned, we identified a large cohort (*n* = 661) of patients with interfering dystonic and hypokinetic‐rigid motor subtypes which were excluded from our analysis. Former research might have implemented these patients in their analysis which might explain the different effects regarding onsets.

Former research described dystonia as a symptom which is related to increased HD stages with longer motor disease duration (Zande et al., 2017). In our large cohort, dystonic patients were younger than predominant choreatic and hypokinetic‐rigid patients (all *p* < .001). Additionally, if compared to choreatic subjects (*p* = .054), the disease duration of dystonic participants did not differ. Hence, we assumed that dystonia may occur early and is not solely accompanied with longer disease durations, which is unexpected and partly in opposite to earlier findings.

Moreover dystonia seems to have an early impact since patients and families notice symptoms earlier than in the choreatic group (all *p* < .001). These results go along with dystonia research data additionally describing patients suffering from pain and emotional harm caused by dystonia with a high impact on the quality of life (Bernstein et al., 2016; Torres & Rosales, 2017).

Regarding most tasks for cognition and TMS, there was no difference between the dystonic and the choreatic group, except for SDMT which might be due to a motor impairment and verbal fluency (180 s) for letters. Dystonic patients showed better cognitive performance in all categories when compared to hypokinetic‐rigid patients (all *p* < .001).

Comparison of TFC was different in all groups, whereas dystonic patients ranged in‐between choreatic with higher and hypokinetic‐rigid with lower capacities. Thus, recent research describing greater impact of dystonia and rigidity compared to chorea on functionality can be confirmed in a large cohort (Carlozzi et al., 2019).

Group differences were found for CAG repeats, disease duration, education, TMS, and CAP score, with lower CAG repeats in the choreatic group versus the hypokinetic‐rigid (*p* < .001) but also for the choreatic versus the dystonic group (*p* < .001). Since differences might have an impact, we focused on controlling these which resulted in no change in findings for cognitive and functional capacities. Thus, group differences are not because of underlying effects from varying covariates.

Apart from an increased apathy sum score in the hypokinetic‐rigid group versus the dystonic and choreatic group, we did not observe any differences in the severity of psychiatric symptoms between groups. Besides, less executive function was observed in the hypokinetic versus the choreatic group (PBA score). This is remarkable because patients with focal dystonia complained about increased psychiatric symptoms with depression, anxiety, and obsessive‐compulsive disorders (Jahanshahi, 2017). We therefore initially hypothesize that dystonia may lead to more depression in HD which is however not supported by our data. Hereby, unawareness in HD patients might be a possible explanation (Hergert, Haaland, & Cimino, 2019; Hergert, Sanchez‐Ramos, & Cimino, 2019; McCusker & Loy, 2014; Sitek, Thompson, Craufurd, & Snowden, 2014). According to our data, functional status and cognition seem not primary influenced by psychiatric status.

## 5 CONCLUSION

In summary, we were able to confirm better cognitive capacity of choreatic versus hypokinetic‐rigid patients and extend this knowledge to dystonic patients showing a cognitive capacity similar to choreatic. In this study, we identified and characterized a large cohort of dystonic HD patients for the first time. Motoric phenotypes are relevant for functional and cognitive capacities independent from described co‐variates. On the one hand, dystonic patients seem to have a similar onset to hypokinetic‐rigid patients, but on the other hand, a cognitive performance is similar to choreatic patients, which is not explained by higher CAG repeats, CAP scores, age, disease duration, or education.

## CONFLICT OF INTEREST

Jannis Achenbach, Sarah Maria von Hein, and Dr. Carsten Saft reported no conflict of interest that relate to the research covered in this article.

## AUTHORS' CONTRIBUTION

Jannis Achenbach involved in conception, organization, and execution of research project; design and execution of statistical analysis; writing of the first draft of manuscript. Sarah Maria von Hein involved in conception of research project; review and critique of statistical analysis; review and critique of manuscript. Carsten Saft involved in conception and organization of research project; review and critique of statistical analysis; review and critique of manuscript.

## Data Availability

The data that support the findings of this study are available from the corresponding author upon request.
